# Valproic acid induces histologic changes and decreases androgen receptor levels of testis and epididymis in rats

**Published:** 2017-04-10

**Authors:** Sitthichai Iamsaard, Wannisa Sukhorum, Supatcharee Arun, Nichapa Phunchago, Nongnuch Uabundit, Porntip Boonruangsri, Malivalaya Namking

**Affiliations:** 1 *Department of Anatomy, Faculty of Medicine, Khon Kaen University, Khon Kaen, Thailand.*; 2 *Center for Research and Development of Herbal Health Products, Faculty of Pharmaceutical Sciences, Khon Kaen University, Khon Kaen 40002, Thailand.*; 3 *School of Medicine, Mae Fah Luang University, Chiang Rai 57100, Thailand.*

**Keywords:** Valproic acid, Testis, Epididymis, Histopathology, Androgen receptor

## Abstract

**Background:**

Valproic acid (VPA), an anti-epileptic drug, can cause male subfertility. However, the degree to which testicular and epididymal histopathologies and androgen receptor (AR) expression are changed under VPA treatment has never been reported.

**Objective:**

To investigate the histopathological changes and AR protein levels of testis and epididymis in VPA-treated rats for every single day.

**Materials and Methods:**

Sixty-four adult male Wistar rats were divided into control and VPA-treated groups (n=8/ each). Treated rats were injected with 500 mg/ kgBW, intraperitoneally, VPA for 10 consecutive days. At the end of every experimental day, all reproductive parameters including histology by hematoxylin and eosin staining and protein expression of AR by Immuno-Western blot in testis and epididymis were examined.

**Results:**

VPA-treated rats showed dramatically changes in testicular and epididymal histopathologies compared to control group. The multinucleated giant cells and sloughing of germ cells were observed on day 6. The germ cell disintegration and increased intercellular spaces of seminiferous tubular epithelium appeared in days 7-10 of VPA treatment. Additionally, extensive multinucleated giant cells and complete exfoliation were clearly found from days 8-10. Such exfoliated germ cells were clearly seen in its epididymal lumen at day 10. The increasing rate of sperm concentration was approximately 32.31% of that in control group at day 10 (*p*=0.03). Moreover, the protein expressions of testicular and epididymal AR (% intensity/ 80 µg protein lysate) was decreased in VPA-treated rats compared with control.

**Conclusion:**

VPA treatment induces histologic changes of germ cell epithelium in seminiferous tubules and decreases the expression of testicular and epididymal androgen receptors.

## Introduction

Although valproic acid (VPA), an anticonvulsant drug, is widely used for treatments in many neurological disorders especially epilepsy and anticancer activities, side effects on the gastrointestinal, neurological, hematological, endocrine, and reproductive systems have been documented (1-6). Indeed, VPA decreases the fertile reproductive parameters and hormones in both epileptic men and experimental animals (4, 5). It has been reported that VPA-administration causes atrophy of the testis, epididymis, prostate gland, and seminal vesicles (9, 10). The testicular atrophy has been shown to be associated with adverse reproductive parameters and decreased sex hormone levels (11-17).

However, the dynamic events showing seminiferous germ cell epithelial damages have never been demonstrated. In biochemical changes of the VPA-treated testis, increased levels of malondialdehyde and decreased antioxidant activities were investigated (12, 16). Recently, our previous results have demonstrated that VPA increased precocious sperm acrosome reactions and induced fibrosis of the tunica albuginea and tubule basement membrane in rat testis (18). Moreover, the expressions of testicular Ki-67, cytochrome P450scc, and phosphorylated proteins (41, 51, and 83 kDas) were significantly decreased in VPA-treated rats (18). 

However, the molecular mechanism underlying the effects of VPA on the male reproductive system is still required. In this regard, it is well known that androgen receptor (AR) classified as a member of the nuclear receptor superfamily is localized in testis and epididymis playing important roles in male spermatogenesis and fertility (19, 20). Generally, AR also is a ligand-dependent transcription factor that regulates the expression of many androgen-responsive genes. However, the sequent changes of reproductive histopathology and the protein expression levels of AR in testis and epididymis in VPA treatment have never been documented. 

Therefore, the aim of this study was to investigate the dynamic changes of testicular and epididymal histopathology together with their AR expressions in VPA-treated rats.

## Materials and methods

**Animals and VPA treatment**

In this experimental animal study, adult Wistar rats (200-230 gr, n=64) were purchased from the National Laboratory Animal Center (Salaya, NakhonPathom, Thailand). Animals were housed within in polycarbonate cages (constant temperature (22±2^o^C) under 12-hr light/dark cycles), in the North-east Laboratory Animal Center, KhonKaen University. All rats received the commercial pellet diet and water ad libitum. Rats were divided into control and VPA-treated (days1-10) groups (n=8/each). Rats in the control group were injected intraperitoneally (i.p.) with normal saline, while those animals of in the experimental group were injected with a single dose of VPA (Sigma-Aldrich, Inc., USA) at 500 mg/kg BW per day for 10 consecutive days as described previously (12, 17, 18). At the end of experiment daily, all rats in each different day (1-10) were anesthetized with sodium pentobarbital (35 mg/kg, i.p.) and euthanized by cervical dislocation. 

**Morphological examinations**

After euthanasia, the right testes and epididymis plus vas deferens of all rats were collected and weighted. Gross morphology of the testis was observed and captured by using a digital camera (Sony Cyber short 7.2 mega pixel cameras, Sony Japan). Such organs from each animal group were fixed in 10% formalin in phosphate-buffered saline (pH 7.4) for 48 hr and processed using routine using paraffin-embedding methods. 

Afterward, the paraffinized-tissue blocks were sectioned at a 5-7μm thickness (Semi-automatic Rotary Microtome, ERM 3100 Semi-Automatic Microtome; Hestion, Australia). All sections were stained with haematoxylin and eosin (H&E) to observe histopathological changes daily. All histological images were photographed at different magnifications using a Nikon light ECLIPSE E200 microscope equipped with a DXM1200 digital camera (Nikon, Japan). 

**Sperm count**

The left caudal epididymis and vas deferens were operated and squeezed to collect sperm fluid. Then, sperm fluid was dipped and suspended in 1 ml of phosphate buffer saline (PBS, 37^o^C, pH=7.4). To wash and separate the mature sperm pellet from its fluid, subsequently, the diluted sperm suspension was centrifuged at 5,000 rpm for 2 min. The re-suspended sperm suspension was subsequently diluted with PBS (1:20 dilution) before counting. The diluted sperm suspension (10 µl) was laid on the Neubauer counting chamber. Then, the sperm was counted under a light microscope (Nikon ECLIPSE E200, Japan) in triplicate examinations as described previously (18, 21).

**Tissue protein preparation and Western blot analysis**

The total protein concentration of the testicular or epididymal supernatant was measured by using a NanoDrop ND-1000 Spectrophotometer (NanoDrop Technologies, Inc., USA) at an absorbance of 280 nm. Such tissue protein lysates (150 μg) were separated by 10% SDS-PAGE. Then, the separated proteins were transferred on to nitrocellulose membranes. Subsequently, each membrane was incubated with a 5% skim-milk blocking solution (Fluka®, USA) for 2 h at room temperature. After washing with 0.05% PBST (PBS, 0.05% (v/v) Tween-20), the blotted membranes were further incubated with anti-androgen receptor (N-20) antibody (1: 200 (v/v); Santa Cruz Biotechnology, Inc., USA) at 4^o^C for overnight. 

Membranes were then washed and incubated with an HRP-conjugated secondary antibody (goat anti-rabbit IgG, Invitrogen™, USA) for 1 hr at room temperature. Membranes were then washed for three times with 0.05% TBST. The β-actin was used as internal control. The level of specific protein expression in tissues was detected using an enhanced chemiluminescence substrate reagent kits (GE Healthcare Life Sciences, USA) under GelDoc 4 imaging system (Image Quant 400; GH Healthcare Life Sciences, USA). 

**Ethical consideration**

This study was approved by Animal Ethics Committee of KhonKaen University, based on the Ethics of Animal Experimentation of National Research Council of Thailand (Rec. No. ACUC-KKU-23/2559, ref no. 0514.1.75/30).

**Statistical analysis**

To evaluate the significance of differences between two groups using Statistical Package for the Social Sciences, version 19.0, SPSS Inc, Chicago, Illinois, USA (SPSS), all parameters were initially subjected to the Shapiro-Wilk test (*W*-test) to confirm normal distribution and equality of variances. Independent Student’s *t*-tests (used for analyzing of normally distributed data) or the Mann-Whitney *U*-test (used for non-normally distributed data) was applied used to compare mean values for the all parameters analyzed. A Two-sided p<0.05 was considered as significant difference. All data were expressed as the mean±SD.

## Results

**Effect of VPA on the body and male reproductive organ weights**

The results showed that the increase of body weights in VPA-treated day 10 was approximately 45.04% as compared to the control day 10 (p=0.01, Table I). In absolute weight of reproductive organs, testis, epididymis plus vas deferens, and seminal vesicle plus prostate gland of the VPA-D10 group were significantly decreased compared to control-D10 group (p*=*0.03). In the same vein, their relative weights except for epididymis plus vas deferens in VPA-D10 rats were significantly decreased compared with those of control-D10 rats (p=0.03).

**Effect of VPA on morphology of rat testes and sperm concentration**

**Figure 1:**
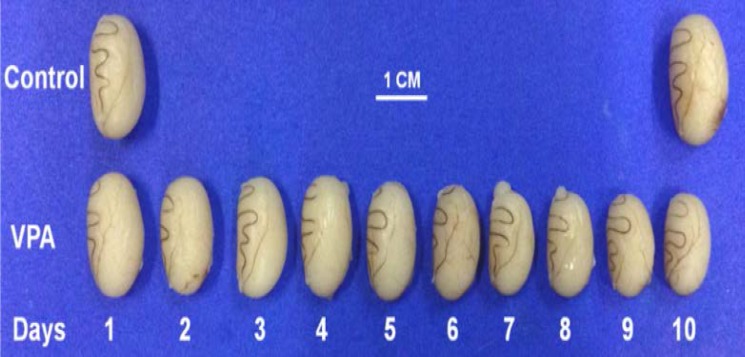
Comparative morphology of rat testes between the controls and VPA-treated groups (days 1-10)

**Figure 2:**
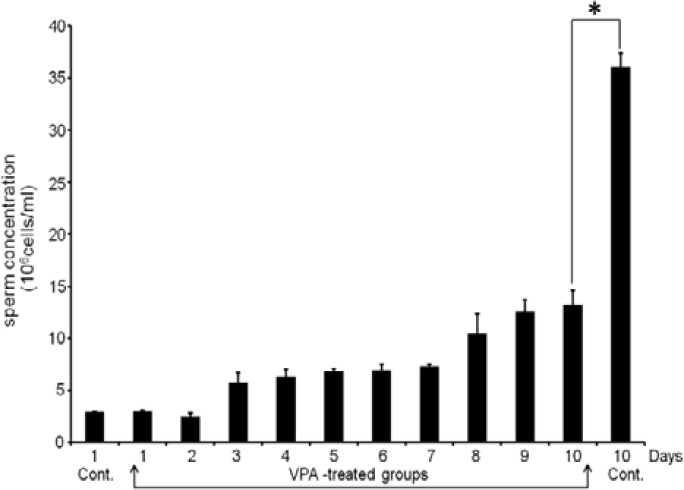
The sperm concentration of rats treated with VPA (500mg/kgBW) for consecutive 10 days compared to controls (days 1 - 10)

In corresponding to absolute weight of testis, figure 1 shows that the testicular size of VPA-treated groups is dramatically decreased from day 5-10. Compared to control day 10 (Figure 1), the testicular size in VPA-treated day 10 (height, 1.36±0.01 cm, width 0.90±0.02 cm) is significantly smaller than that of control (height 1.75±0.02 cm; width 1.13±0.01 cm). As shown in figure 2, the increasing rate of epididymal sperm concentration in VPA-day 10 group was approximately 32.31% of that in control day 10 group (p*=*0.03, significantly different).

**Effect of VPA on testicular histology**

**Figure 3:**
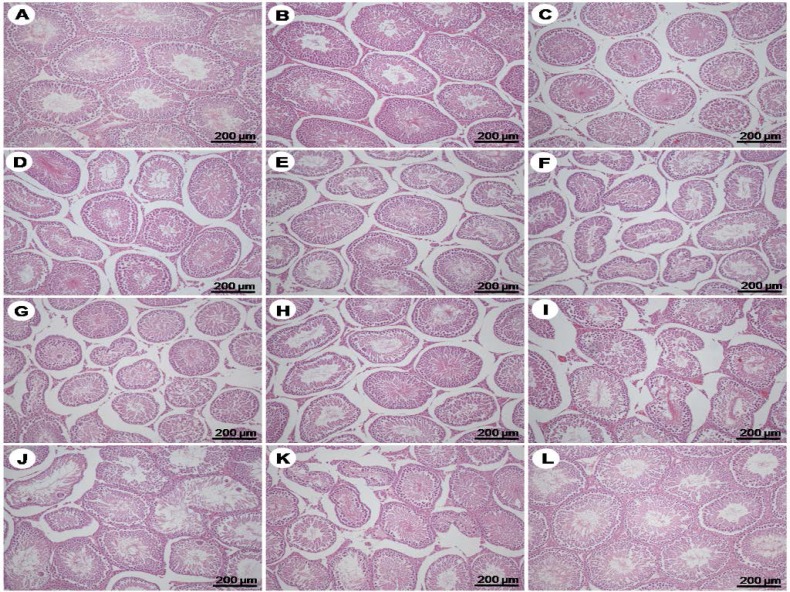
Photomicrographs showing histology of testis stained by H&E of the control day1 (A), VPA treated days 1-10 (B-K), and control day 10 (L), respectively

**Figure 4:**
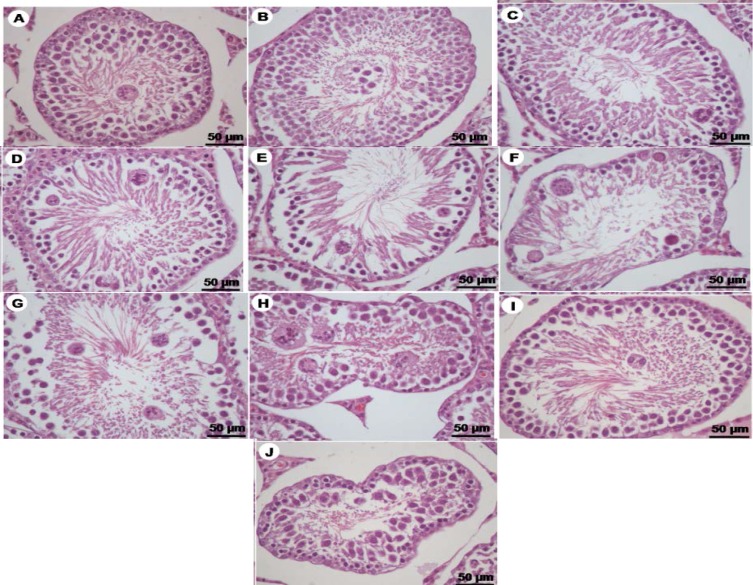
Photomicrographs showing histopathology of seminiferous tubules stained by H&E of VPA-treated day 6(A+B), 7(C+D), 8(E+F), 9(G+H), and 10(I+J), respectively

The testicular histology in all experimental groups were shown in figure 3. It found that the shrinking of seminiferous tubules began to be seen at day 2 of VPA treatment (Figure 3). Obviously, the increased interstitial spaces were found from days 2-10 but the Leydig cells still remained in the interstitium (Figure 3C-4K). The severity degrees of atrophic tubules were investigated from days 4 to 10 (Figure 3E-K). In addition, none of the character cells of spermatogenesis such as multinucleated giant cells appeared in some tubules of VPA-treated day 6-10 (Figure 3G-K).

**Histopathology of seminiferous tubules and epididymis induced with VPA**

**Figure 5:**
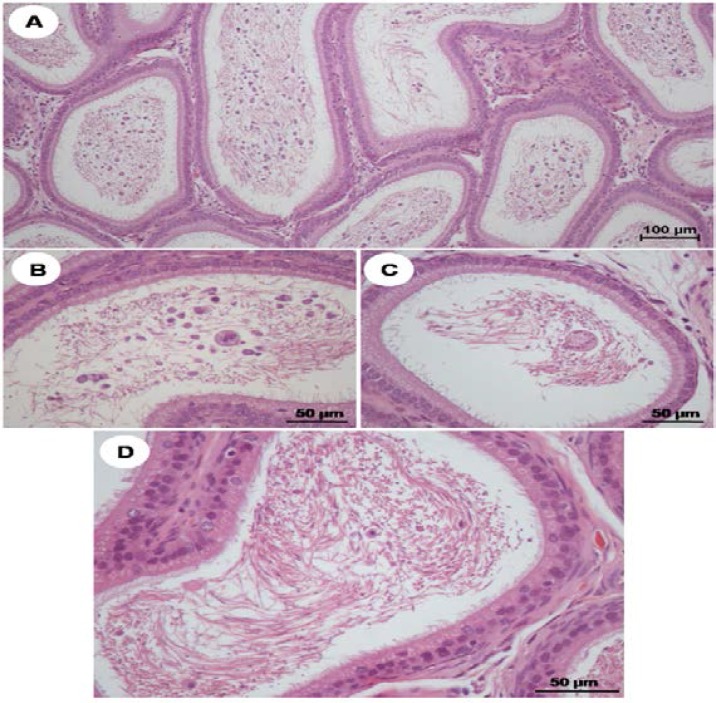
Photomicrographs showing histopathology (H&E) of caput epididymis (A-C) and cauda epididymis (D) of VPA-treated day10

The histopathology of seminiferous tubules were exhibited in days 6-10 of VPA-treated groups are shown in figure 4A-J. The multinucleated giant cells and sloughing of germ cells were found in day 6 (Figure 4 and 5). The day 7-10, VPA induced disintegration and increased intercellular spaces of seminiferous tubular epithelium (Figure 4C-J). It seems to be found chromatin condensation, nucleus pyknosis, and increased eosinophilia of apoptotic germ cells in days 7-9 (Figure 4C-H). Additionally, extensive nucleus of multinucleated giant cells and complete exfoliation were clearly investigated from days 8-10 (Figure 4E-J). Figure 5 showed that an abundance of exfoliated germ cells was seen in the lumen of the epididymis of D10-VPA treated rats. It was noted that the caput epididymis of D10-VPA treated group contained numbers of extensively round and multinucleated giant cells with low sperm mass (Figure 5A-C). Interestingly, the extent of such abnormal cells was obviously less in cauda epididymis of VPA-treated day 10 (Figure 5D). However, epithelium of caput plus cauda epididymis treated with VPA looked normal (Figure 5).

**Effect of VPA on level of androgen receptor expression**

**Figure 6:**
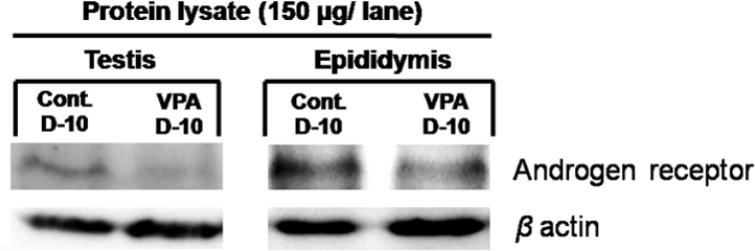
Representative immuno-Western blotting of androgen receptor in testicular and epididymal protein lysate of control and VPA groups taken from experiment day 10 (n = 4). β-actin was used as internal control

In immuno-Western blotting of the testicular and epididymal lysates taken from day 10 of the experiment, the representative results showed that the levels of androgen receptor protein expression in such reproductive tissues were lower in VPA-treated rats as compared to the control (Figure 6).

**Table I:** The body and male reproductive organ weights of rats treated with VPA for 10 consecutive days compared to the controls

**Groups**	**Body weight (g)**	**Absolute weight (g)**			**Relative weight (g/100 g)**		
		**Testis**	**Epididymis plus vas deferens**	**Seminal vesicle plus prostate gland**	**Testis**	**Epididymis plus vas deferens**	**Seminal vesicle plus prostate gland**
Cont-D1	254.65±4.67	1.36±0.07	0.23±0.02	0.42±0.05	0.54±0.03	0.09±0.01	0.17±0.02
VPA-D1	249.99±5.41	1.36±0.02	0.25±0.03	0.38±0.02	0.57±0.02	0.10±0.01	0.16±0.01
VPA-D2	245.06±6.77	1.24±0.05	0.25±0.02	0.42±0.08	0.51±0.05	0.10±0.01	0.17±0.04
VPA-D3	246.04±13.03	1.29±0.05	0.26±0.02	0.44±0.08	0.53±0.03	0.11±0.01	0.18±0.03
VPA-D4	247.19±11.73	1.29±0.10	0.28±0.03	0.47±0.07	0.49±0.02	0.11±0.01	0.18±0.02
VPA-D5	252.06±19.15	1.19±0.10	0.26±0.03	0.45±0.12	0.47±0.01	0.10±0.01	0.18±0.04
VPA-D6	255.64±16.07	1.17±0.14	0.27±0.03	0.45±0.4	0.46±0.03	0.11±0.01	0.18±0.04
VPA-D7	257.16±15.16	1.16±0.16	0.27±0.02	0.55±0.06	0.43±0.06	0.11±0.02	0.21±0.02
VPA-D8	245.63±10.15	1.16±1.17	0.30±0.02	0.53±0.11	0.43±0.04	0.12±0.01	0.22±0.04
VPA-D9	256.60±14.45	1.16±0.04	0.32±0.02	0.56±0.13	0.46±0.01	0.13±0.01	0.22±0.04
VPA-D10	272.11±8.86*	1.14±0.09*	0.32±0.01*	0.66±0.06*	0.42±0.01*	0.12±0.00	0.24±0.02*
Cont-D10	303.76±9.01	1.64±0.08	0.37±0.02	1.15±0.22	0.54±0.04	0.12±0.00	0.38±0.06

D: day

VPA: valproic acid

Cont: control.

* Significant differences (P<0.05), compared with the control-D10 group. Data are represented as means±SD (n=8 / each group)

## Discussion

This study attempted to demonstrate the histopathology of testis and epididymis of rats treated with VPA (500 mg/kgBW) for 10 consecutive days in dynamic event for the first time. Consequently, VPA induced seminiferous tubular atrophy, germinal cellular sloughing, and multinucleated giant cell formation, respectively. Indeed, that testicular histopathology usually has been shown at day 10 after VPA treatment as previously reported (12, 16-18, 22). In addition, the results showed that such degenerated germ cells were abundantly observed in caput epididymis and declined in cauda epididymis of day 10. This finding strongly suggested that epididymis plays an important role in phagocytosis of immature germ cells (23, 24). These classical features of testicular damages have been shown in many reports using other chemical inductions (25-28).

However, the actual mechanism underlying such gametotoxicity is still unknown. In common, the germinal sloughing is explained to be a result of the damage to Sertoli cells and interruption of intergerminal bridge. Similar to animal treated with different chemicals rats, VPA might induce the formation of multinucleated giant cells (also called symplasts) via fusion of damaged round spermatids (27). These cells were known to appear in the final common pathway of germinal cell degeneration although their formations are still unclear. It was hypothesized that multinucleated giant cells result from karyokinesis which not followed by cytoplasmic division (27). AR has been localized in both testis and epididymis (29-31). 

The expression of AR is directly related to androgen-available circulating of male reproductive system. Via androgen actions, it is well known that AR is essentially responsible for normal development and function of postnatal testis and epididymis (32). Many reports have shown that some drugs or chemicals can induce reproductive organ atrophy that is associated with decreased AR expression (32-34). As demonstrated in this study that VPA could induce testicular atrophy by damaging of seminiferous epithelium (Figs 3&4), this effect might be associated with the decreased level of AR (Figure 6). 

This association seemed similar to other reports showing the interruption of the circulating androgen availability with lower AR expression resulting in reproductive atrophy (32-34). It is also assumed that VPA might affect the AR-depending genes responsible for the translation of many proteins important for spermatogenesis and testosterone synthesis. A previous study clearly showed that the expression levels of steroidogenic acute regulatory (StAR), cytochrome P450 side-chain cleavage (CYP11A1), Ki67, and phosphorylated proteins in testis were significantly altered under 10 day-VPA treatment (18). However, gene expressions of such proteins need to be further elucidated to clarify their relations to the decreased levels of AR protein expression as shown in this study.

## Conclusion

This study demonstrated that VPA could induce histopathology of germ cell epithelium in seminiferous tubules starting at day 6 and the exfoliated germ cells including cell debris were accumulated at caput epididymis at day 10 of VPA-treated rats. VPA also decreased the levels of the AR expression in testis and epididymis. 
